# Supplementary figures and images for: Superagers Resist Typical Age-Related White Matter Structural Changes
Source: J Neurosci. 2024 Apr 29;44(25):e2059232024. doi: 10.1523/JNEUROSCI.2059-23.2024 (PMC11209667; doi:10.1523/JNEUROSCI.2059-23.2024)

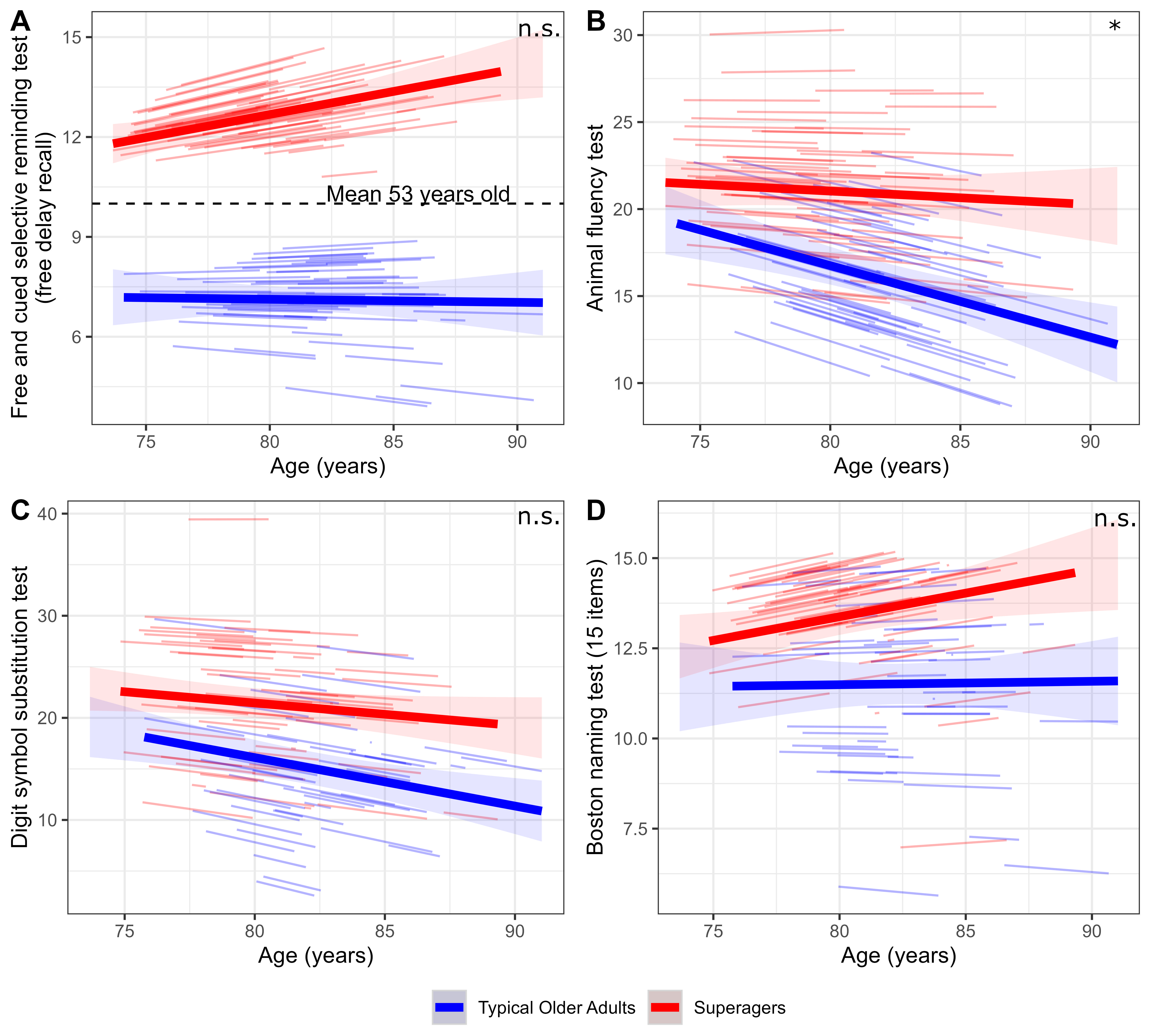

Supplement: Table 1-1 — Longitudinal trajectories of the neuropsychological tests used for selection criteria. Linear fits of performance trajectories of A. the free and cued selective reminding test raw free delayed recall score, B. the animal fluency test total score, C. the digit symbol substitution test total score and D. the 15-item Boston naming test total score over time are plotted for superagers (red solid line) and typical older adults (blue solid line). Respective shaded areas indicate the 95% confidence interval and individual participant trajectories are also plotted (thin lines). The threshold for episodic memory performance in superagers (at or above the mean score of a 53-year-old person with the same education level) is indicated with a dashed line in the free and cued selective reminding test plot. For the rest of the tests, typical older adults did not have a set criterion while superagers had to perform within one standard deviation from the mean for their age and education. In linear mixed effects models assessing group differences in longitudinal neuropsychological performance, age was scaled but raw values are shown for illustration purposes. The significant interaction between scaled age and group (P < 0.05) is indicated with an asterisk (*), otherwise as non-significant (n.s.). Download Table 1-1, TIF file. [file jneuro-44-e2059232024-s001.tif]

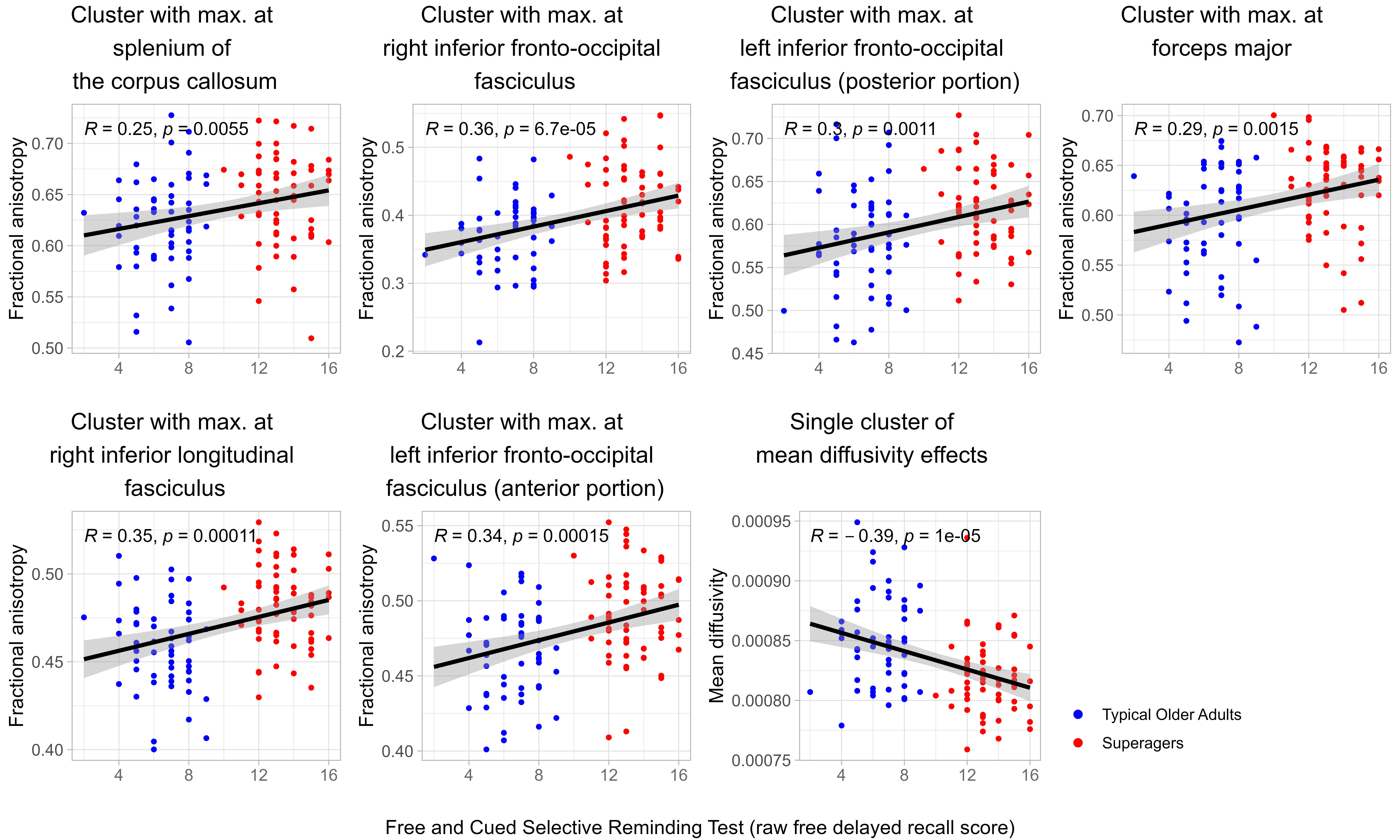

Supplement: Figure 1-1 — Association between fractional anisotropy and mean diffusivity values and episodic memory performance in superagers and typical older adults. Fractional anisotropy and mean diffusivity values were extracted from the cross-sectional diffusivity images averaging the values of the significant clusters from group contrasts, six clusters from the contrast higher fractional anisotropy in superagers vs. typical older adults and a single large cluster form the contrast higher mean diffusivity in typical older adults vs. superagers. The correlation with the raw free delayed recall score of the free and cued reminding test was performed with a Peason’s correlation test and the correlation coefficient (R) and the p-value (p) of each test are shown in the plot. The clusters are named under the white matter tract of the JHU-ICBM atlas where their global maximum is located. Download Figure 1-1, TIF file. [file jneuro-44-e2059232024-s002.tif]

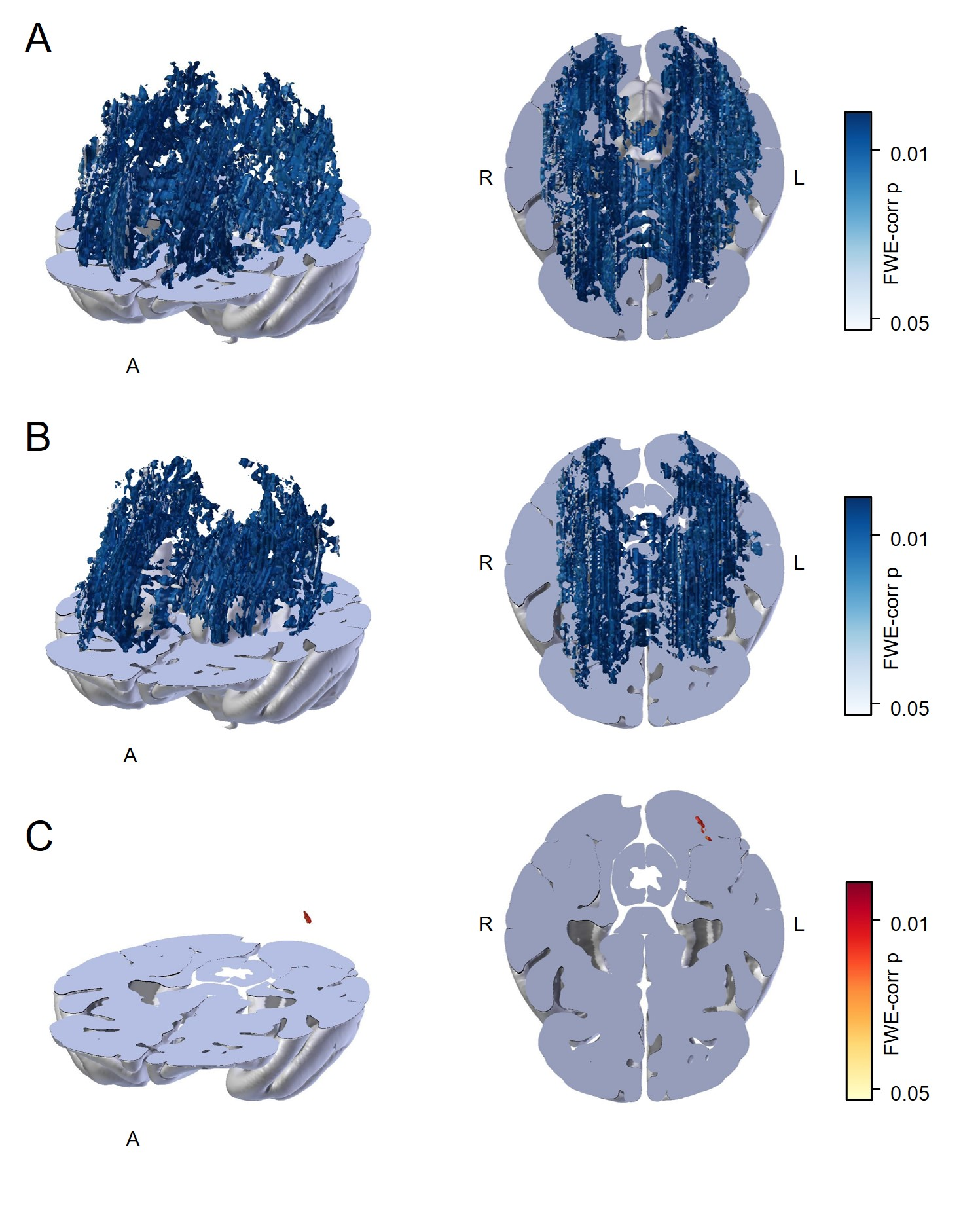

Supplement: Figure 1-2 — Radial and axial diffusivity and mode of anisotropy cross-sectional differences between superagers and typical older adults. A. Lower radial diffusivity and B. lower axial diffusivity is found in superagers compared to typical older adults in an extensive network (cold colours) comprising all the tracts described in JHU-ICBM atlas consisting with the group effects in mean diffusivity. C. Higher mode of anisotropy is found in superagers than typical older adults in a small part of the left inferior longitudinal fasciculus and forceps major (warm colours). This result shows that, despite the group differences in fractional anisotropy that reflect a stronger directionality of water diffusivity in the frontal tracts of superager’s brains, there is no major group differences in the shape of this directionality. (P < 0.05 FWE-corrected). A, anterior; FWE-corr p, family-wise error p-value; L, left; R, right and P, posterior. Download Figure 1-2, TIF file. [file jneuro-44-e2059232024-s003.tif]

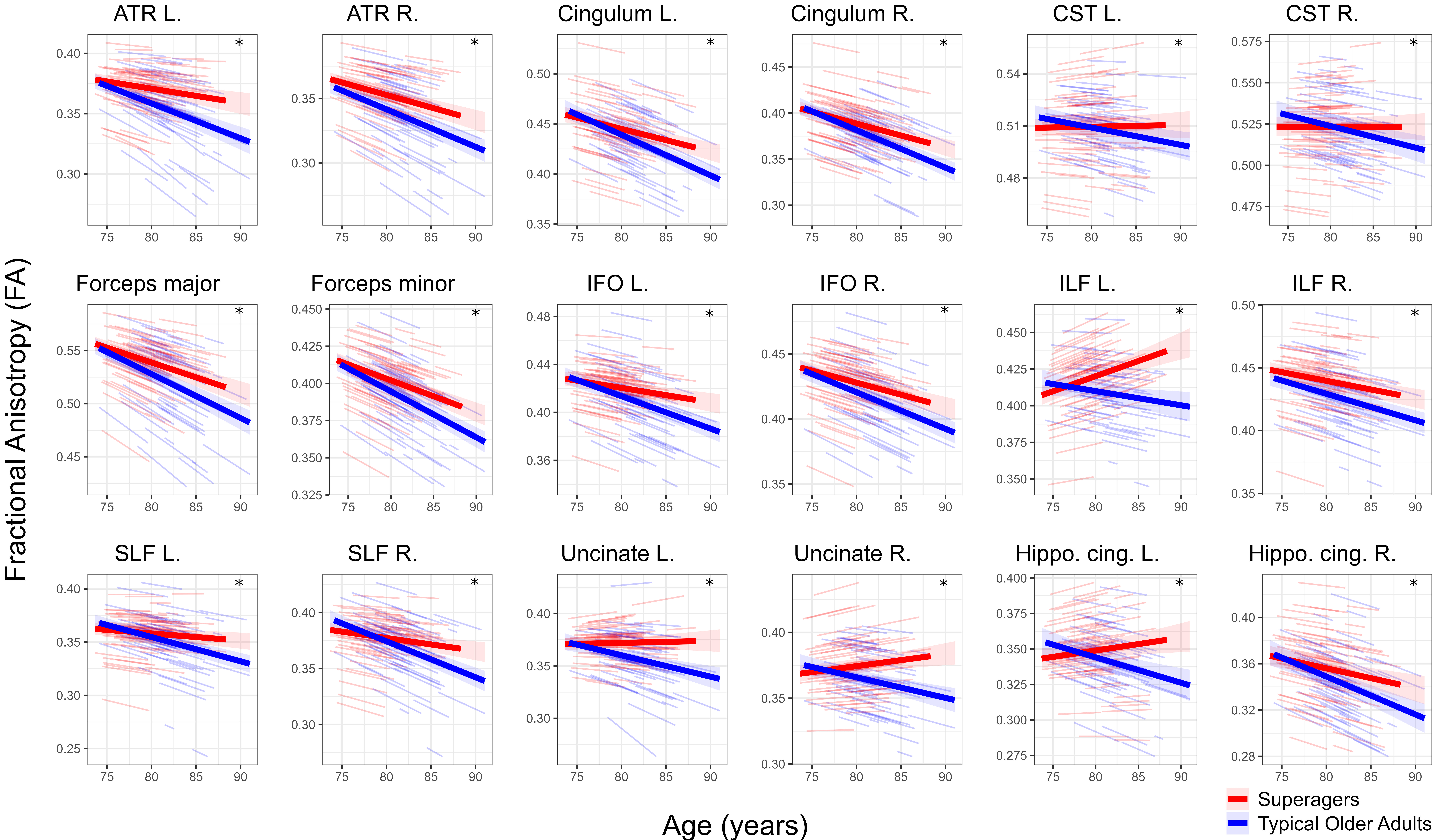

Supplement: Figure 3-1 — ROI-based longitudinal trajectories of white matter fractional anisotropy (FA). Longitudinal group differences were studied in 18 white matter tracts or regions of interest (ROIs) from the JHU-ICBM atlas. Individual trajectories are plotted together with the group average predicted trajectory. Shaded areas indicate 95% confidence interval. A linear mixed effects model was used to predict average FA in each of the ROIs with group, scaled age and the interaction between the two as fixed factors, the random intercept and slope were included in the model. Age was scaled in the statistical model, but raw values are shown for illustration purposes. All ROIs show a significant (P < 0.05) interaction between age and group (indicated with an asterisk (*)). ATR, anterior thalamic radiation; CST, corticospinal tract; Hippo. cing., hippocampal cingulum; IFO, inferior fronto-occipital fasciculus; ILF, inferior longitudinal fasciculus; L, left; R, right; SLF, superior longitudinal fasciculus. Download Figure 3-1, TIF file. [file jneuro-44-e2059232024-s005.tif]

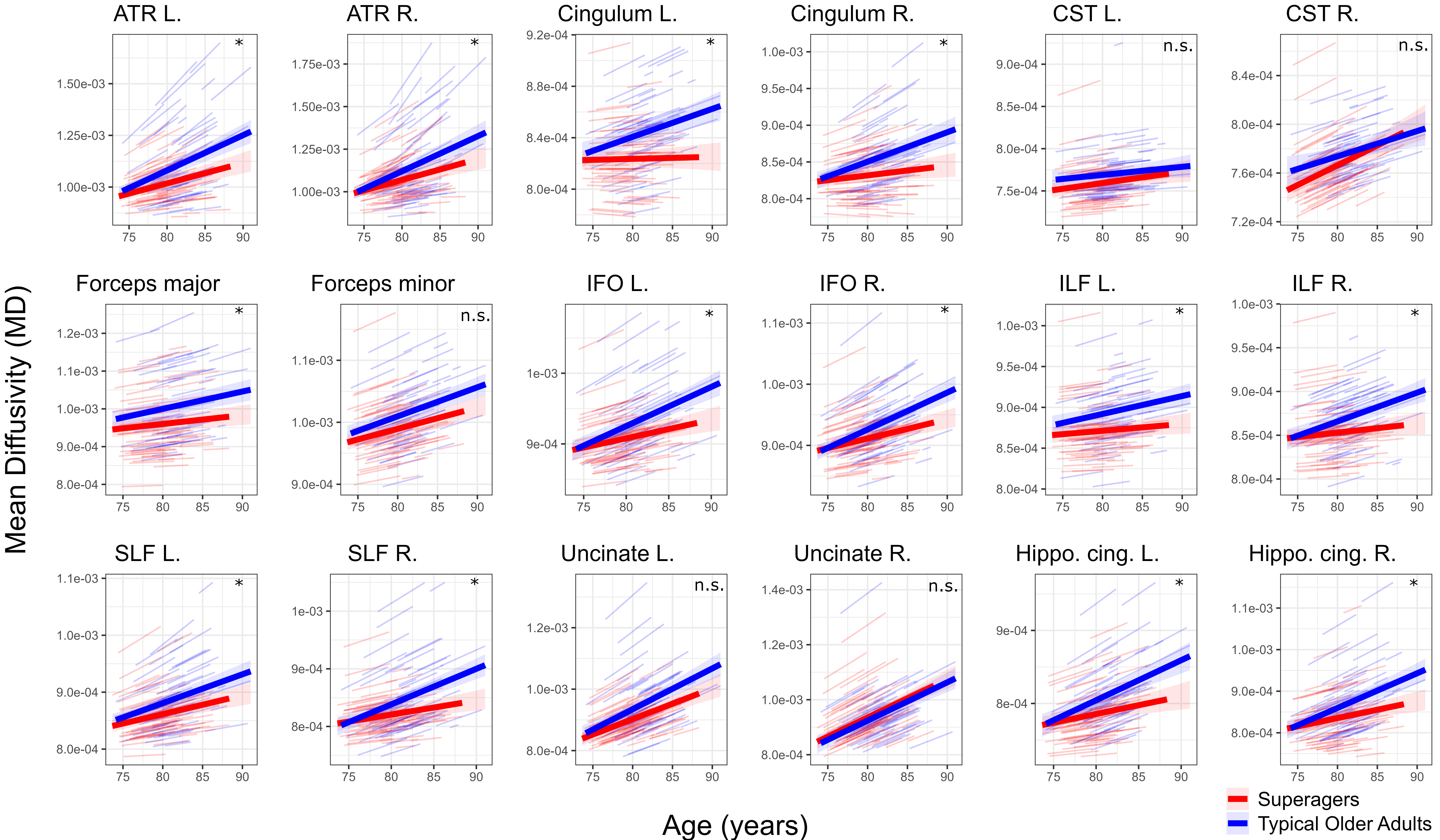

Supplement: Figure 3-3 — ROI-based longitudinal trajectories of white matter mean diffusivity (MD). Longitudinal group differences were studied bilaterally in 18 white matter tracts or regions of interest (ROIs) from the JHU-ICBM atlas. Individual trajectories are plotted together with the group average predicted trajectory. Shaded areas indicate 95% confidence interval. A linear mixed effects model was used to predict average MD in each of the ROIs with group, scaled age and the interaction between the two as fixed factors, the random intercept and slope were included in the model. Age was scaled in the statistical model, but raw values are shown for illustration purposes. ROIs where the interaction between age and group is significant (P < 0.05) are indicated with an asterisk (*), otherwise as non-significant (n.s.). ATR, anterior thalamic radiation; CST, corticospinal tract; Hippo. cing., hippocampal cingulum; IFO, inferior fronto-occipital fasciculus; ILF, inferior longitudinal fasciculus; L, left; R, right; SLF, superior longitudinal fasciculus. Download Figure 3-3, TIF file. [file jneuro-44-e2059232024-s007.tif]
