# Supplementary material for: Superagers Resist Typical Age-Related White Matter Structural Changes
Source: J Neurosci. 2024 Apr 29;44(25):e2059232024. doi: 10.1523/JNEUROSCI.2059-23.2024 (PMC11209667; doi:10.1523/JNEUROSCI.2059-23.2024)
Supplement: Figure 2-1 — Longitudinal evolution of white matter volume, white matter lesions volume and Fazekas score. Coefficients (β) correspond to the three linear mixed effects model predicting the longitudinal evolution of total brain white matter volume, brain white matter lesion volume and Fazekas score progression respectively. In the three independent models, age, group and the interaction between age and group were fixed effects (scaled age was introduced in the model) and the random intercept and slope were also considered in the model. In the white matter lesions volume analysis four outliers were excluded (three typical older adults and a superager). White matter volume and white matter lesions volume were adjusted by total intracranial volume (TIV). P, p-value; SD, standard deviation; SE, standard error. Download Figure 2-1, DOCX file. [file jneuro-44-e2059232024-s004.docx]

|  | White matter volume  (TIV-adjusted) | | White matter lesions volume (TIV-adjusted) | | Fazekas score | |
| --- | --- | --- | --- | --- | --- | --- |
|  | **β (SE)** | ***P*** | **β (SE)** | ***P*** | **β (SE)** | ***P*** |
| **Group** | -4.62 (6.11) | 0.45 | 0.38 (0.47) | 0.42 | -0.07 (0.14) | 0.63 |
| **Age (scaled)** | -18.58 (1.13) | < 0.0001 | 0.69 (0.14) | < 0.0001 | 0.10 (0.04) | 0.007 |
| **Group x Age** | 0.40 (1.73) | 0.81 | 0.33 (0.21) | 0.11 | 0.01 (0.05) | 0.80 |
| **Superager slope**, cm^3^/one SD of scaled age | -18.58 (1.13) | - | 0.69 (0.14) | - | 0.11 (0.03) | - |
| **Typical older adult slope**, cm^3^/one SD of scaled age | -18.18 (1.30) | - | 1.01 (0.15) | - | 0.10 (0.04) | - |
