# Supplementary material for: Superagers Resist Typical Age-Related White Matter Structural Changes
Source: J Neurosci. 2024 Apr 29;44(25):e2059232024. doi: 10.1523/JNEUROSCI.2059-23.2024 (PMC11209667; doi:10.1523/JNEUROSCI.2059-23.2024)
Supplement: Figure 3-4 — ROI-based longitudinal analysis of white matter mean diffusivity (MD). Longitudinal group differences were studied in 18 white matter tracts or regions of interest (ROIs) from the JHU-ICBM atlas with a linear mixed effects model to predict average MD in each of the ROIs with group, scaled age and the interaction between the two as fixed factors, the random intercept and slope were included in the model. ATR, anterior thalamic radiation; β, coefficients; Corr. P, false-discovery-rate corrected p-value; CST, corticospinal tract; Hippo. cing., hippocampal cingulum; IFO, inferior fronto-occipital fasciculus; ILF, inferior longitudinal fasciculus; L, left; P, p-value; R, right; SE, standard error; SLF, superior longitudinal fasciculus. Download Figure 3-4, DOCX file. [file jneuro-44-e2059232024-s008.docx]

|  | Group | | | Age (scaled) | | | | Group x age (scaled) | | | | Slope superagers | | Slope typical older adults | |  |
| --- | --- | --- | --- | --- | --- | --- | --- | --- | --- | --- | --- | --- | --- | --- | --- | --- |
|  | β (SE) | *P* | Corr. *P* | | β (SE) | *P* | Corr. *P* | | β (SE) | *P* | Corr. *P* | | estimate (SE) | | estimate (SE) | |
| **ATR L.** | 6.7x10^-5^ (2.5x10^-5^) | 0.007 | 0.02 | | 3.1x10^-5^ (4.9x10^-6^) | 5.1x10^-10^ | 2.9x10^-9^ | | 2.3x10^-5^ (7.4x10^-6^) | 0.002 | 0.001 | | 3.1x10^-10^ (4.9x10^-11^) | | 5.3x10^-10^ (5.5x10^-11^) | |
| **ATR R.** | 5.7x10^-5^ (3.0x10^-5^) | 0.06 | 0.07 | | 3.8x10^-5^ (6.1x10^-6^) | 4.3x10^-10^ | 2.9x10^-9^ | | 2.6x10^-5^ (9.2x10^-6^) | 0.004 | 0.01 | | 3.9x10^-10^ (6.2x10^-11^) | | 6.5x10^-10^ (6.8x10^-11^) | |
| **Cingulum L.** | 1.9x10^-5^ (5.1x10^-6^) | 0.0002 | 0.004 | | 8.2x10^-8^ (1.4x10^-6^) | 0.95 | 0.95 | | 6.7x10^-6^ (2.2x10^-6^) | 0.002 | 0.001 | | 2.0x10^-12^ (1.4x10^-11^) | | 6.7x10^-11^ (1.6x10^-11^) | |
| **Cingulum R.** | 2.1x10^-5^ (7.0x10^-6^) | 0.003 | 0.02 | | 3.9x10^-6^ (1.8x10^-6^) | 0.03 | 0.04 | | 8.7x10^-6^ (2.8x10^-6^) | 0.002 | 0.01 | | 4.0x10^-11^ (1.8x10^-11^) | | 1.3x10^-10^ (2.1x10^-11^) | |
| **CST L.** | 9.0x10^-6^ (5.5x10^-6^) | 0.10 | 0.11 | | 4.3x10^-6^ (1.5x10^-6^) | 0.005 | 0.007 | | -1.0x10^-6^ (2.4x10^-6^) | 0.66 | 0.70 | | 4.3x10^-11^ (1.5x10^-11^) | | 3.2x10^-11^ (1.8x10^-11^) | |
| **CST R.** | 6.0x10^-6^ (4.8x10^-6^) | 0.21 | 0.22 | | 1.0x10^-5^ (1.8x10^-6^) | 1.3x10^-8^ | 3.9x10^-8^ | | -3.5x10^-6^ (2.8x10^-6^) | 0.21 | 0.25 | | 1.0x10^-10^ (1.8x10^-11^) | | 6.4x10^-11^ (2.1x10^-11^) | |
| **Forceps major** | 4.1x10^-5^ (1.6x10^-5^) | 0.01 | 0.03 | | 6.7x10^-6^ (2.1x10^-6^) | 0.002 | 0.003 | | 8.0x10^-6^ (3.2x10^-6^) | 0.01 | 0.02 | | 6.7x10^-11^ (2.1x10^-11^) | | 1.5x10^-10^ (2.5x10^-11^) | |
| **Forceps minor** | 2.1x10^-5^ (8.9x10^-6^) | 0.02 | 0.03 | | 1.1x10^-5^ (1.7x10^-6^) | 8.0x10^-10^ | 2.9x10^-9^ | | 4.1x10^-6^ (2.7x10^-6^) | 0.12 | 0.16 | | 1.1x10^-10^ (1.7x10^-11^) | | 1.5x10^-10^ (2.0x10^-11^) | |
| **IFO L.** | 1.9x10^-5^ (7.9x10^-6^) | 0.02 | 0.03 | | 7.6x10^-6^ (2.9x10^-6^) | 0.009 | 0.01 | | 9.0x10^-6^ (4.4x10^-6^) | 0.04 | 0.06 | | 7.7x10^-11^ (2.9x10^-11^) | | 1.7x10^-10^ (3.2x10^-11^) | |
| **IFO R.** | 1.7x10^-5^ (8.2x10^-6^) | 0.04 | 0.05 | | 9.6x10^-6^ (1.6x10^-6^) | 7.0x10^-10^ | 2.9x10^-9^ | | 9.1x10^-6^ (2.4x10^-6^) | 0.0001 | 0.002 | | 9.7x10^-11^ (1.5x10^-11^) | | 1.9x10^-10^ (1.8x10^-11^) | |
| **ILF L.** | 2.2x10^-5^ (6.7x10^-6^) | 0.0009 | 0.008 | | 2.2x10^-6^ (1.5x10^-6^) | 0.13 | 0.14 | | 4.8x10^-6^ (2.3x10^-6^) | 0.03 | 0.05 | | 2.3x10^-11^ (1.5x10^-11^) | | 6.9x10^-11^ (1.7x10^-11^) | |
| **ILF R.** | 1.5x10^-5^ (6.2x10^-6^) | 0.02 | 0.03 | | 3.2x10^-6^ (1.6x10^-6^) | 0.05 | 0.06 | | 7.0x10^-6^ (2.5x10^-6^) | 0.005 | 0.01 | | 3.2x10^-11^ (1.6x10^-11^) | | 1.0x10^-10^ (1.9x10^-11^) | |
| **SLF L.** | 2.1x10^-5^ (8.7x10^-6^) | 0.02 | 0.03 | | 1.0e-05 (1.9e-06) | 6.8x10^-8^ | 1.8x10^-7^ | | 5.6x10^-6^ (2.9x10^-6^) | 0.05 | 0.07 | | 1.0x10^-10^ (1.9x10^-11^) | | 1.6x10^-11^ (2.2x10^-11^) | |
| **SLF R.** | 2.1x10^-5^ (8.5x10^-6^) | 0.02 | 0.03 | | 7.0x10^-6^ (3.0x10^-6^) | 0.02 | 0.03 | | 1.2x10^-5^ (4.5x10^-6^) | 0.006 | 0.01 | | 7.2x10^-11^ (3.0x10^-11^) | | 1.9x10^-10^ (3.3x10^-11^) | |
| **Uncinate L.** | 3.7x10^-5^ (1.5x10^-5^) | 0.02 | 0.03 | | 3.2x10^-5^ (6.2x10^-6^) | 2.1x10^-7^ | 4.7x10^-7^ | | 1.0x10^-5^ (9.3x10^-6^) | 0.26 | 0.29 | | 3.2x10^-10^ (6.2x10^-11^) | | 4.2x10^-10^ (6.9x10^-11^) | |
| **Uncinate R.** | -1.1x10^-5^ (1.8x10^-5^) | 0.55 | 0.55 | | 4.3x10^-5^ (4.0x10^-6^) | <2.0x10^-16^ | 3.6x10^-15^ | | 9.6x10^-7^ (6.1x10^-6^) | 0.88 | 0.88 | | 4.3x10^-10^ (4.0x10^-11^) | | 4.4x10^-10^ (4.6x10^-11^) | |
| **Hippo. cing. L.** | 2.1x10^-5^ (7.5x10^-6^) | 0.005 | 0.02 | | 7.6x10^-6^ (2.3x10^-6^) | 0.001 | 0.002 | | 9.8x10^-6^ (3.6x10^-6^) | 0.007 | 0.01 | | 7.9x10^-11^ (2.3x10^-11^) | | 1.7x10^-10^ (2.7x10^-11^) | |
| **Hippo. cing. R.** | 2.7x10^-5^ (1.2x10^-5^) | 0.03 | 0.04 | | 1.3x10^-5^ (3.1x10^-6^) | 5.0x10^-5^ | 1.0x10^-4^ | | 1.3x10^-5^ (4.8x10^-6^) | 0.005 | 0.01 | | 1.3x10^-10^ (3.1x10^-11^) | | 2.6x10^-10^ (3.6x10^-11^) | |
